# Variational Principle of Least Psychomotor Action: Modelling Effects on Action from Disturbances in Psychomotor Work Involving Human, Cyborg, and Robot Workers

**DOI:** 10.3390/e21060543

**Published:** 2019-05-28

**Authors:** Stephen Fox, Adrian Kotelba

**Affiliations:** VTT Technical Research Centre of Finland, FI-02044 VTT, Finland

**Keywords:** artificial intelligence, autonomous, craft: cyber-physical systems, cyborg, digitalization, human, industrial, manual work, Markov chains, microstates, perturbation theory, psychomotor, robot, situated entropy, skills, work, worker

## Abstract

Optimal psychomotor work can be expressed in terms of the principle of least psychomotor action (PLPA). Modelling psychomotor action encompasses modelling workers, work, and interactions between them that involve different types of situated entropy. Modelling of psychomotor workers encompasses three types of workers: human, cyborg, and robot. The type of worker and the type of work interact to affect positioning actions, performing actions, and perfecting actions undertaken in psychomotor tasks. There are often disturbances in psychomotor work, for example due to weather conditions, which have a determining influence on what work can be undertaken with least psychomotor action by different types of workers. In this paper, findings are reported from a study focused on the modelling disturbances in psychomotor work. Five contributions are provided. First, a heuristic framework for modelling disturbances and their effects is provided. In addition to PLPA and situated entropy, this framework encompasses Markov processes, the theory of perturbations, and calculus of variations. Second, formulae and ratios are provided for heuristic modelling of effects on internal action (*S_int_*) from disturbances to psychomotor work. Third, formulae and ratios are provided for heuristic modelling of effects on external action (*S_e_*). Fourth, examples are provided of heuristic modelling of disturbances in psychomotor work. Fifth, formulae and examples show how task complexity can be modelled heuristically in terms of microstates across the cyber domain and the physical domain of cyber-physical systems. Overall, the study reported in this paper addresses variational aspects of PLPA.

## 1. Introduction

Disturbances in production work have been modelled previously in manufacturing [1,2] and in construction [3,4]. However, previous modelling has been concerned with autonomous systems [1,2,3,4]. Rather than disturbances in production that involves interactions between different types of work and different types of workers. Our own previous work has encompassed different types of work and different types of workers, but not disturbances in production [5,6]. In this paper, five contributions are made to address this shortcoming in the previous work. First, a heuristic framework for modelling disturbances and their effects is provided. The heuristic framework encompasses Markov processes, the theory of perturbations, and calculus of variations. Second, formulae and ratios are provided for heuristic modelling of effects on internal action (*S_int_*) from disturbances to psychomotor work. Third, formulae and ratios are provided for heuristic modelling effects on external action (*S_e_*). Fourth, examples are provided of heuristic modelling of disturbances in psychomotor work. Fifth, examples show how task complexity can be modelled heuristically in terms of microstates across the cyber domain and the physical domain of cyber-physical systems. Together, these contributions enable heuristic modelling of effects from disturbances on interactions between diverse psychomotor work and workers: rather than on autonomous systems [1,2,3,4].

The study reported here builds upon research reported in two previous papers in Entropy. The first paper [5] provided an explanation of how resources for physical production work, such as work instructions, product components, and workstations, can be carriers of situated information, and also carriers of various types of situated entropy. The second paper [6] expanded upon this [5] by generalizing from examples to the three categories of work setting, work composition, and work uncertainty, and to three aspects of worker action: positioning, performing, and perfecting. In addition, details were provided about the state-of-the-art for psychomotor capabilities of human, cyborgs, and robot workers. Moreover, the principle of least psychomotor action (PLPA) was introduced as follows: the preferred combination of worker types is that which can carry out psychomotor work with the least internal action (*S****_int_***) and least external action (*S**_e_***). Here, cyborgs are humans who are enhanced by permanent implanting or persistent wearing of work technologies [7,8,9,10,11,12,13]. As shown in Table 1, implanting or wearing of work technologies, such as exoskeletons, can introduce new sources of disturbances and affect the action required to carry out work.

Together with references to relevant theory [14,15,16,17], Table 1 provides a summary of variables and examples of disturbances in psychomotor work. For example, there can be disturbances arising from workers affected by fatigue, exoskeleton misalignment, sensor error, etc. Also, disturbances can arise from work settings amongst erratic weather conditions, from work compositions that include natural materials with unique grain patterns, and from work characterized by the uncertainty of customer-led design, such as inconsistent interfaces between one-of-a-kind components. Instances of such disturbances are bounded, and conform to conceptualization of disturbances in systems theory. In particular, low frequency of recurrence, low temporal predictability, and production of significant deviation from normal state [18,19].

The remainder of the paper comprises five further sections. Next, in Section 2, the heuristic framework is introduced. Then, in Section 3 and Section 4, formulae and ratios are introduced for heuristic modelling of *S_int_* and for heuristic modelling of *S_e_*. Subsequently, in Section 5, examples are provided. In conclusion, principal findings, implications, limitations, and directions for further research are discussed in Section 6.

## 2. Heuristic Framework for Modelling Disturbances in Psychomotor Work

### 2.1. Rule-of-Thumb Heuristics

The duality of entropy across the cyber domain and physical domain supports rule-of-thumb heuristic structuring of complexity modelling in cyber-physical systems involving humans, cyborgs, and robots [20,21]. Rules-of-thumb are widely used for heuristic structuring of complex problems in many sectors [22,23]. They are consistent with the scientific preference for simplicity [24], and allow rapid comparisons of options to be carried out where there is incomplete information [25]. As well as enabling decisions to be made quickly with limited information, rule-of-thumb heuristics avoid overfitting. That is avoid the production of an analysis that corresponds too exactly to a particular set of data, and may therefore fail to fit additional data or predict future observations reliably [26,27]. Rule-of-thumb heuristics are widely used across science [28,29,30] and engineering [31,32,33], including to address diverse non-trivial problems involving measurement [34,35,36]. Rule-of-thumb heuristics are appropriate in engineering design when there are several alternative options to be considered, and the exact performance of each option cannot be measured accurately in advance [37,38,39,40]. Thus, a rule-of-thumb framework is appropriate for comparative evaluation of alternative options for combining humans, cyborgs, and robots in production work. Moreover, rules-of-thumb heuristics are applied successfully in strategic decision-making [41] and capital investment appraisal [42]. Hence, a rule-of-thumb heuristic framework is appropriate where modelling will be used to inform strategic decision making, which can involve potentially large capital investments in robotics and computer-integrated manufacturing.

Entropy is well-suited to rule-of-thumb heuristic modelling of work complexity. For example, comparison of alternative plans for carrying out production work is straightforward. In particular, the worst plan is the plan with highest entropy and the best plan is the plan with lowest entropy across all of the tasks to be carried out. Also, the entropy of each alternative for carrying out a task can be measured individually, and then the entropy of all tasks can be added together to provide total entropy for overall production plans. Thus, alternative production plans can be analyzed in detail. Furthermore, logarithms are used in calculation of entropy so probability distributions for microstates across many variables do not escalate into unmanageably huge numbers [20,21,43]. As explained in the following sections, in-keeping with the fundamental requirement for simplicity in rule-of-thumb heuristics, we combine widely applied scientific constructs and entropy mathematics in a simple framework. The explanation is thorough in comprising 43 formulae, but is distilled into three main formulae, (25), (28), and (43), three rule-of-thumb ratios, and one rule-of-thumb construct for expressing task complexity in the cyber domain in the physical domain. Together, from robust scientific foundations we provide a novel heuristic framework for modelling psychomotor complexity and corresponding action. As shown in the examples in Section 5, implementation of the heuristic framework is straightforward and requires no digital simulations or other sophisticated electronic modelling tools. Rather, it involves application of novel rule-of-thumb heuristics, which are in-keeping with the scientific preference for simplicity [24] and allows rapid comparisons of options to be carried out where there is incomplete information [25].

### 2.2. States in Psychomotor Work: Flow and Choke

Two states are particularly relevant to psychomotor work: flow and choke. Internal action (*S_int_*) and external action (*S_e_*) can be merged together (*S*) in the flow of autonomous action. Disturbances interrupt flow and choke autonomous action. This leads to there being less coupling between *S_int_* and *S_e_* as workers stop to think or compute what they are going to do in order to deal with the disturbance [44]. For example, human fatigue can lead to human workers being clumsier, wearing of standard exoskeletons for non-standard work can lead to misalignments that affect cyborg balance, and sensor errors can lead to counterproductive robot action selection. Work issues, such as bad weather, unpredictable material properties, and inconsistent component interfaces, can combine with worker issues to increase the potential for disturbances, such as falling on wet ground, unintentionally damaging natural materials, and counterproductive actions in fabricating component interfaces. It is important to note that it is often easier to go from old sources of disturbances rather than to eliminate disturbances completely. For example, the soft folds of a veil can be formed around the face of a marble statue through hand carving [45]. However, disturbances can be common because the marble has its own unique natural characteristics, which the carver must continually try to anticipate throughout this delicate psychomotor work. Such disturbances to psychomotor work can be eliminated by producing a geometrically identical statue from synthetic powders using three-dimensional additive manufacturing (e.g., 3D printing). However, 3D printing involves interactions between materials and processes that can lead to disturbances that lead to rework that involves psychomotor actions [46].

Flow involves carrying out many external actions (*S_e_*) with little, or no, internal action (*S_int_*) [6,44]. In psychomotor production work, trial-and-error actions can involve initial *S_i_* about what to do followed by much *S_e_*, which due to automaticity, is driven by little internal action until what is being trialed is produced sufficiently to be judged for its fitness for purpose. Automaticity is evolved in human workers from the basis of general psychomotor abilities, including kinesthetic integration, manual dexterity, physical balance, and spatial perception. Fine and gross psychomotor abilities can be combined with little, if any, conscious thought; for example, when carrying work tools up the irregular slopes of a hillside. General psychomotor abilities involve embodied cognition enabled by innate human attributes, such as proprioception and neural suppression. Proprioception involves unconscious sensing of relative positions of different parts of the body during movement. Neural suppression is a brain process that automatically encourages selection of well-worn neural pathways. For example, adult human beings do not have to think about how to walk, because thousands of previous walking steps have established well-worn neural pathways in the brain that make physical walking an automatic psychomotor ability [16,47,48,49].

Typically, adult human beings have vast repertoires of general psychomotor abilities acquired through daily life, play, sports, etc. [50]. Acquisition of psychomotor skills can take place from the base of general psychomotor abilities in three stages: cognitive, associative, and autonomous. During the cognitive stage, the learner becomes cognitively aware of the demands of the skill to be learnt. The associative stage involves practice and feedback. Next, the learner may advance to the autonomous stage of being able to perform the skill elegantly with minimal cognitive effort [51]. Eventually, the learner may become able to transfer psychomotor skills successfully to new tasks in new settings [52,53,54]. The higher the mastery of psychomotor skills, the less thought (*S_int_*) is involved in their performance [55]. For example, Sugar Ray Robinson, who is widely rated as the world’s best ever boxer and who went 91 boxing matches undefeated, is quoted as saying that after sufficient training, “You do not think. It is all instinct. If you stop to think, you are gone” [56].

### 2.3. Perturbation Theory and Disturbances in Psychomotor Work

Perturbation theory involves applying known solutions for related simpler problems to perturbed problems. This involves considering the problem as having characteristics that are the same as the simpler problem and also having characteristics that are perturbed. This involves progressively more refined orders of approximation. For example, a first approximation of planetary motions can encompass one planet and the Sun moving in Kepler’s orbits. The approximation from two planetary bodies can become more refined by encompassing three planetary bodies, and so on. The last solution obtained from application of perturbation theory may still be only approximate but nonetheless enables problem solving. For example, the planet Neptune was discovered through application of perturbation theory [57,58].

Problem solving with perturbation theory is analogous with trial-and-error disturbance solving in psychomotor work when the trial is informed by the known solutions provided by existing templates for general psychomotor abilities and existing schema for psychomotor work skills. As summarized in Table 2, there are different levels of known solutions that can be applied in trial-and-error. For example, at the general level, there can be templates for psychomotor abilities to walk, stand, and sit in order to do work, and at the work skill level there can be schema for reforming interfaces between products. The known solutions of schema and templates provide internal models [59,60]. In particular, forward internal models that enable predictive simulation of sensory consequences of an action [61,62]. Application of known solutions to perturbed problems in planetary motion and to disturbances in psychomotor work involves iterations of parameter estimation—first to identify what known solutions to apply and then to identify to that which is outside the scope of the known solutions applied first [63,64].

In Table 2, the known solutions of work skills schema are for rework required to address disturbances in psychomotor work.

**Figure 1:**
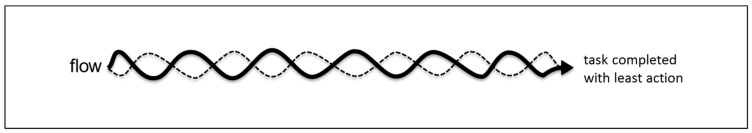
Flow state of autonomous psychomotor action.

As illustrated in Figure 1, work that is not disturbed can be carried out in the autonomous flow of least action where there is more *S_e_* (solid line) than *S_int_* (dotted line) due to automaticity [44,51,52,53,54,55,56].

**Figure 2:**
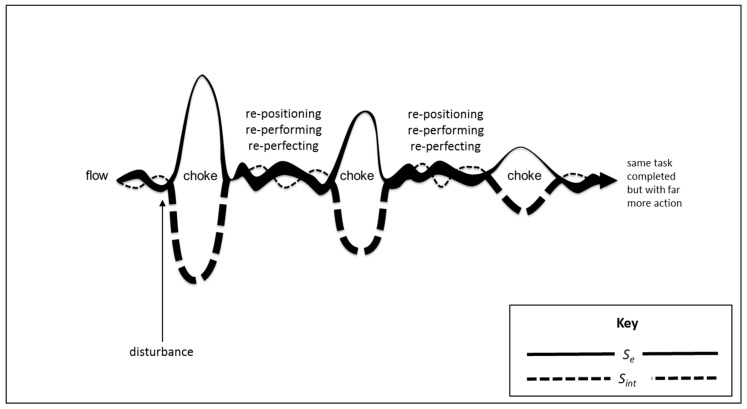
The flow of autonomous psychomotor action choked following disturbance.

In contrast, as illustrated in Figure 2, one disturbance can lead to several chokes in the flow of autonomous action. This is because the worker needs to engage in iterations of conscious thought or computational effort for parameter estimation and known solution selection. As shown in Figure 2, *S_int_* (dotted line) is highest during these iterations when the worker has to think or compute what to do next.

The first choke shown in Figure 2 arises from the disturbance and identifying what known solutions to apply to the disturbed task. This is followed by increased *S_e_* (solid line). There is increased *S_e_* because of two reasons. First, additional positioning actions (i.e., re-positioning actions) are needed to deal with a disturbance before being able to undertake performing actions [65,66]. Second, there are additional performing actions in the form of rework (i.e., re-performing) [67]. For example, a disturbance involving a bad cut into a wooden component with a coarse cutting tool leads to rework with finer cutting tools. This may involve comparatively little *S_int_*, because much of such remedial work can be done with automaticity. For example, comparatively little *S_int_* is required to perform the repetitive motion of rubbing down damaged wooden components with sand paper, but plenty of *S_e_* is involved. Third, re-perfecting may involve some additional external action; for example, in repeating a motion in order to commit it to memory.

Overall, *S_e_* depends upon the complexity of work arising from the disturbances. As described in more detail in Section 4 and Section 5, complexity is modelled in terms of number of microstates. Correspondence between modelling of information and of mechanics in terms of microstates has already been defined by others [22,68], and has the advantage of enabling modelling across the cyber domain and physical domain of cyber-physical systems involving different types of workers [69,70]. 

The subsequent two choke-points shown in Figure 2 arise from identifying what known solutions to apply to the work that still remains to be done because the first known solution applied was not sufficient to complete the disturbed task. The more iterations there are, the more potential there is for *S_e_* (solid line) to increase through additional biomechanical motion or robot mechatronic motion carried out with automaticity. Thus, energy expenditure increases during completion of the disturbed task, which takes place over a longer period of time.

### 2.4. Two-State Markov Processes

**Figure 3:**
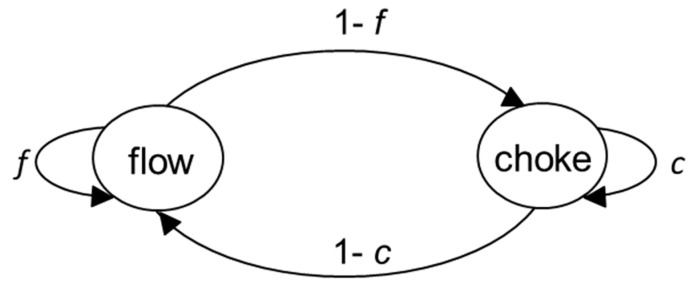
Flow and choke states in psychomotor work.

As summarized in Figure 3, we consider two principal possible states of the production system comprising of work and workers: flow and choke.

We assume that the probabilities of occurrence of particular conditions are known a priori or can be reliably estimated. Then, the probabilities of transiting from one state to another, given the conditions in the preceding time instance, can be represented by a transition matrix

(1) $$P=\left[\begin{array}{cc} p_{11} & p_{12}\\ p_{21} & p_{22} \end{array}\right]=\left[\begin{array}{cc} f & 1-f\\ 1-c & c \end{array}\right]$$

In other words, the matrix P represents the model, in which a flow state is 100 *f* per cent likely to be followed by another flow state, and a choke is 100 *c* likely to be followed by another choke state. The columns of the matrix P can be labelled flow and choke, and the rows can be labelled in the same order. More specifically, the entry $p_{ij}$ of the matrix P is the probability that if a given state is of type *i*, it will be followed by a state of type *j*. A stationary distribution of a Markov process is represented as a row vector ρ, and given transition matrix P, it satisfies

(2) ρ=ρP

In other words, ρ is invariant by the matrix P. Let $\rho_{f}$ denote the stationary probability of being in flow state and $\rho_{c}$ denote the stationary probability of being in choke state [71]. Then,

(3) $$\rho_{f}=\frac{1-c}{1-c+1-f}$$

and

(4) $$\rho_{c}=\frac{1-f}{1-c+1-f}$$

Finally, we assume that the initial state of a Markov process is drawn according to the stationary distribution ρ, and thus the Markov process is a stationary process [71].

Changes in *S_int_* due to disturbances can be modelled using entropy for sum of a random variable *N* and some function g(*X*) of a random variable *X*. More specifically, random variable *X* denotes the solution obtained in a “normal” workflow, transformation g:X→G denotes deterministic part of the worker’s response to disturbance, and random variable *N* denotes residual disturbance. Thus, under disturbances, we use differential entropy *h*(*G+N*) rather than *h*(*X*). The total variation can be applied in order to model biomechanical motion or robot mechatronic motion (*S_e_*) in terms of how much executed action for the disturbed task differs from the optimum least action of the undisturbed task. The unit of *S_e_*, and of the first variation, is the product of energy and time. Additional energy expenditure can be obtained by dividing the total variation by execution time [72,73]. This modelling is applied in the following section for positioning action, performing action, and perfecting action.

## 3. Heuristic Modelling of Effects on Internal Action (*S_int_*) from Disturbances

In this section, formulae and ratios are provided for modelling effects on internal action (*S_int_*) from disturbances. Heuristic ratios are applied widely in planning of production work [74,75], including for characterizing effects on production from the different attributes of different worker types [76,77] to determine the most suitable mix of worker types [78,79]. The ratios stated in this section are novel, as they are for different effects on *S_int_* in production work due to different attributes of human, cyborg, and robot workers.

### 3.1. Formulae

Psychomotor work involves positioning, performing, and perfecting actions [6]. Let us denote positioning actions by *X*, performing actions by *Y*, and perfecting actions by *Z*. The total situated entropy *h* can be represented as joint entropy of random variables modeling positioning, performing, and perfecting steps, or by chain law of entropy

(5) h(X, Y, Z)=h(X)+h(Y|X)+h(Z|Y,X).

We model the complexity of a given task using a conditional entropy *h*(*Y*|*X*). Modelling of positioning *h*(*X*) and perfecting *h*(Z|Y,X) aspects of psychomotor work is more complex. Hence, Friston’s information-based model is drawn upon to describe quantitatively positioning and perfecting aspects of psychomotor work [80,81].

With regard to positioning actions, for *S_int_*, suppose that worker takes position *x* after reception of sensory input *r* under a model *w* of the world. Then, the informational load of selecting a position (*X*) is measured with conditional entropy

(6) $$h\left(X)=h(R|W\right)=-\iint_{r,w}^{}p\left(r|w\right)p\left(w\right)\log_{2}p\left(r|w\right)drdw.$$

In other words, conditional differential entropy *h*(*R*|*W*) represents average “surprise” of receiving unexpected sensory input in an otherwise known environment. Additional positioning actions (i.e., repositioning actions) are needed to deal with a disturbance before being able to undertake performing actions [82,83].

With regard to performing actions, for *S_int_*, suppose that a worker can be in one of possible positions *X* and that a given task can be performed in one of several possible ways *Y*. Let us denote the current position by *x* and assume that the worker selects position *x* with probability *p*(*x*). Furthermore, let the worker select a certain way to complete the task, denoted by *y*, with probability *p*(*y|x*). Then, the complexity of a given task is measured with the conditional differential entropy

(7) $$h\left(Y|X\right)=-\iint_{x,y}^{}p\left(x\right)p\left(y|x\right)\log_{2}p\left(y|x\right)dxdy.$$

Note that additional performing actions (i.e., rework actions) are needed to deal with a disturbance.

With regard to perfecting actions, for *S_int_*, suppose that a worker receives sensory input *r* as the result of his actions during rework. We assume that *v* is an unknown quantity that caused the sensory state *r* and denote the true distribution of the causes by *q*(*v*|*r*). The worker tries to infer possible cause *v* for the sensory state *r* using their probabilistic representation of the world *μ*. The so-called recognition density *p*(*v*|*μ*) describes the worker’s probabilistic representation of the causes of the sensory inputs. Then, the informational load of perfecting actions is measured with relative entropy

(8) $$h\left(Z|X,Y)=D(p||q\right)=\int_{v}^{}p\left(v|\mu \right)\log_{2}\frac{p\left(v|\mu \right)}{q\left(v|r\right)}dv.$$

The worker can minimize the part of internal action *S_int_* corresponding to perfecting actions by minimizing the relative entropy term *D*(*p*||*q*). This task is usually accomplished by active inference, that is, by optimizing perception and adapting the worker’s recognition density *q*(*v*|*r*) into a better approximation of the true distribution *p*(*v*|*μ*). Also, additional perfecting actions are needed to deal with a disturbance.

Let us now assume that the task is performed under external disturbance, such as those summarized in Table 1, which results in a final position $X^{*}$, performing action $Y^{*}$, and perfecting action $Z^{*}$. In other words, the operator takes a position $X^{*}$ rather than a position X, executes course of action indexed by $Y^{*}$ rather than Y, and perfects the output by performing $Z^{*}$ rather than Z. We assume that the final position $x^{*}$, course of action $y^{*}$, and act of perfecting $z^{*}$ can be modelled as follows:

(9) $$\begin{array}{ccc} x^{*} & = & g_{X}\left(x,y,z\right)+n_{X}\\ y^{*} & = & g_{Y}\left(x,y,z\right)+n_{Y}\\ z^{*} & = & g_{Z}\left(x,y,z\right)+n_{Z} \end{array}$$

where functions $g_{X}:{\mathbb{R}}^{3}\to \mathbb{R}$, $g_{Y}:{\mathbb{R}}^{3}\to \mathbb{R}$, and $g_{Z}:{\mathbb{R}}^{3}\to \mathbb{R}$ are assumed to be differentiable. In words, these functions capture deterministic change in position, course of action, and act of perfecting the outcome due to disturbance. Thus, these functions model operator’s attempt to compensate for disturbance with additional re-positioning, re-performing, and re-perfecting actions. For notational convenience, we write

(10) $$g=g\left(x,y,z\right)=\left[\begin{array}{c} g_{X}\left(x,y,z\right)\\ g_{Y}\left(x,y,z\right)\\ g_{Z}\left(x,y,z\right) \end{array}\right]=\left[\begin{array}{c} x^{*}\\ y^{*}\\ z^{*} \end{array}\right].$$

Note that g is a random vector when (x,y,z) are random. We will assume that random variables $x^{*},\;y^{*}$, and $z^{*}$ have finite variance. In other words, the covariance matrix

(11) $$K_{G}=E\left[GG^{T}\right]$$

of a multivariate random variable *G =* (G_X,_ G_Y,_ G_Z_) has a finite trace. The symbol *E* in Equation (11) denotes expectation.

A random vector

(12) $$n=\left[\begin{array}{c} n_{X}\\ n_{Y}\\ n_{Z} \end{array}\right]$$

in Equation (9) captures random variations in the final position, course of action, and act of perfecting the outcome due to disturbance. Thus, the random vector *n* models the residual disturbance in the outcome that the operator is not able to compensate. Furthermore, we assume that random variables $n_{X}$, $n_{Y}$, and $n_{Z}$ are zero mean random variables and the covariance matrix

(13) $$K_{N}=E\left[NN^{T}\right]$$

of a multivariate random variable *N* = (N_X,_ N_Y,_ N_Z_) *N* has a finite trace. In other words, each of random components$\;n_{X}$, $n_{Y}$, and $n_{Z}$ has a finite variance. We assume that random vectors *G* and *N* are independent.

The total situated entropy under disturbance is

(14) $$h\left(X^{*},Y^{*},Z^{*}\right)=\;h\left(G_{X}+N_{X},G_{Y}+N_{Y},G_{Z}+N_{Z}\right).$$

In general, a closed-form expression for entropy of the sum of random variables is unknown. For that reason, we consider the worst-case and the best-case scenario by introducing, respectively, the upper and the lower bound on the total situated entropy (14).

For random variables with finite variances, the most famous upper bound on Equation (14) is due to Shannon [82]. In particular, let

(15) $$K=E\left[\left(G+N\right){\left(G+N\right)}^{T}\right]$$

denote the covariance matrix of the sum of random variables *G* and *N*. The covariance matrix K is

(16) $$K=K_{G}+K_{N}$$

because *G* and *N* both have finite variance, *G* and *N* are independent, and *N* is zero-mean random variable. Since the maximum entropy distribution under the constraint of a finite trace of ***K*** is a multivariate normal distribution [71], we obtain

(17) $$h\left(X^{*},Y^{*},Z^{*}\right)\leq \frac{1}{2}\log_{2}{\left(2\pi e\right)}^{3}\left|\det\left(K\right)\right|=\;\frac{1}{2}\log_{2}{\left(2\pi e\right)}^{3}\left|\det\left(K_{G}+K_{N}\right)\right|$$

where the symbol detA denotes determinant of a matrix A.

With regard to the lower bound, it is shown in [60] that

(18) $$h\left(X^{*},Y^{*},Z^{*}\right)\geq \frac{1}{2}h\left(G_{X},G_{Y},G_{Z}\right)+\frac{1}{2}h\left(N_{X},N_{Y},N_{Z}\right)+\frac{3}{2}$$

The first part of the lower bound in Equation (18), due to operator’s deterministic response to a disturbance, is

(19) $$h\left(G_{X},G_{Y},G_{Z}\right)=h\left(X,Y,Z\right)+\int_{x,y,z}^{}p_{x,y,z}\left(x,y,z\right)\log_{2}\left|\det\left(J\right)\right|dxdydz\leq \frac{1}{2}\log_{2}{\left(2\pi e\right)}^{3}\left|\det\left(K_{G}\right)\right|$$

where $p_{x,y,z}\left(x,y,z\right)$ denotes the joint probability density function of a random vector (x,y,z), and J is the 3 × 3 Jacobian matrix of partial derivatives given by

(20) $$J=\left[\begin{array}{ccc} \frac{\partial g_{X}}{\partial x} & \frac{\partial g_{X}}{\partial y} & \frac{\partial g_{X}}{\partial z}\\ \frac{\partial g_{Y}}{\partial x} & \frac{\partial g}{\partial y} & \frac{\partial g_{Y}}{\partial z}\\ \frac{\partial g_{Z}}{\partial x} & \frac{\partial g_{Z}}{\partial y} & \frac{\partial g_{Z}}{\partial z} \end{array}\right]$$

The last inequality in Equation (19) follows from the fact that multivariate normal distribution maximizes the entropy for all distributions with the same trace of matrix ***K_G_*** [83]. By the same argument

(21) $$h\left(N_{X},N_{Y},N_{Z}\right)\leq \frac{1}{2}\log_{2}{\left(2\pi e\right)}^{3}\left|\det\left(K_{N}\right)\right|$$

and in order to explicitly include the entropy of undisturbed state, Equation (18) can be rewritten as

(22) $$h\left(X^{*},Y^{*},Z^{*}\right)\geq \frac{h\left(X,Y,Z\right)}{2}+\frac{1}{2}\int_{x,y,z}^{}p_{x,y,z}\left(x,y,z\right)\log_{2}\left|\det\left(J\right)\right|dxdydz+\frac{1}{2}h\left(N_{X},N_{Y},N_{Z}\right)+\frac{3}{2}$$

We have so far demonstrated how to determine the situated entropy of the undisturbed state (5) and the situated entropy of the disturbed state (14). Since the environment evolves between undisturbed and disturbed state, as shown in Figure 3, the final stationary distribution of positioning, performing, and perfecting actions is a mixture of probability distributions p(x,y,z) and $p\left(x^{*},y^{*},z^{*}\right)$ with weights $\rho_{f}$ and $\rho_{c}$, respectively. In general, differential entropy of mixtures does not usually admit closed-form expressions because the log term in entropy definitions transforms into an intractable log-sum term when dealing with mixture densities [84]. However, one can derive the differential entropy when mixture densities are disjointed [71]. In particular, suppose (X,Y,Z) and $\left(X^{*},Y^{*},Z^{*}\right)$ have disjointed support sets and define a new random variable

(23) $$Q=\{\begin{array}{ccc} \left(X,Y,Z\right) & \mathrm{with}\;\mathrm{probability} & \rho_{f}\\ \left(X^{*},Y^{*},Z^{*}\right) & \mathrm{with}\;\mathrm{probability} & \rho_{c} \end{array}$$

Then, a probability density function of *Q* is a mixture density

(24) $$p\left(q\right)=\rho_{f}p\left(x,y,z\right)+\rho_{c}p\left(x^{*},y^{*},z^{*}\right)$$

Provided that mixture densities p(x,y,z) and $p\left(x^{*},y^{*},z^{*}\right)$ are disjointed, by direct application of the definition of differential entropy to (24), we obtain the stationary situated entropy

(25) $$h\left(Q\right)=\rho_{f}h\left(X,Y,Z\right)+\rho_{c}h\left(X^{*},Y^{*},Z^{*}\right)-\rho_{f}\log_{2}\rho_{f}-\rho_{c}\log_{2}\rho_{c}$$

Interestingly, if disturbances are present and mixture densities are disjointed, the situated entropy cannot be reduced to zero by optimizing the production process. This is because the mixing process cannot be controlled by those who engineer production resources to eliminate situated entropy, and so

(26) $$h\left(Q\right)\geq -\rho_{f}\log_{2}\rho_{f}-\rho_{c}\log_{2}\rho_{c}>0$$

when $\rho_{c}>0$.

Furthermore, we assume that the energy used for information processing tasks is proportional to the square of the information complexity of those tasks, which are measured by situated entropy *h* [83,84]. Consequently, the internal action is

(27) $$S_{int}=\int_{t_{1}}^{t_{2}}c_{H}\left(t\right){\left[h\left(t\right)\right]}^{2}dt.$$

where *c_H_*(*t*) denotes the energy cost of processing one bit of information related to complexity of the task, and *h* is provided by Equation (25).

If $c_{H}\left(t\right)$ and h(t) are constant within integration interval $\left(t_{1},t_{2}\right)$, Equation (27) reduces to

(28) $$S_{int}=c_{H}{\left[h\left(Q\right)\right]}^{2}\left(t_{2}-t_{1}\right)$$

### 3.2. Ratios

It is important to note that there are different trade-offs between *c_H_* and *t* for different types of workers. For example, the human brain is more power-efficient than an electronic computer. Yet, the human brain processes information much more slowly than an electronic computer. This trade-off is of fundamental importance because energy is the ability to do work, and energy = power x time. Hence, low power-efficiency can be compensated by high processing speed, and vice versa.

It has been demonstrated in experimental testing of links between information and thermodynamics that the minimum energy cost for processing one bit of information is 0.693 kT joules [85]. However, this is a theoretical absolute minimum rather than a frequent occurrence in practice. Also, it does not distinguish between the means of processing, such as a brain or a computer. In this modelling, we are concerned with differences in power consumption for different types of workers. It has been found that biological brains consume what has been described as “remarkably little power in comparison to electronic computers”, with electronic computers consuming tens of thousands of times more [86]. Interestingly, the human brain consumes little extra power when involved in solving complex problems than when involved in less challenging pastimes. This is because the brain is anyway continually consuming energy in so called “housekeeping” that involves cellular maintenance [87]. However, there are continual efforts to reduce the energy consumption of electronic computation [88]. Accordingly, we assume a conservative power consumption ratio of 1/10,000 for human worker to robot worker in processing one bit of information. Furthermore, as the human brain consumes little more power when involved in more intense mental effort, such as when undertaking a task while wearing an exoskeleton, we assume that the power consumption ratio for the three different types of worker to be 1/1.2/10,000.

On the other hand, electronic computers can undertake computations millions of times faster than the human brain. However, this general advantage in processing times is mediated in psychomotor work by complexity among the individual processing operations to be carried out. In particular, millennia of evolution have enabled the brain to make sense of psychomotor situations with automaticity from comparatively few sensory inputs [46,58]. By contrast, electronic computers are less evolved; hence, the efforts towards brain-inspired computing paradigms [89]. Thus, we assume that the human brain is not millions of times slower than an electronic computer in psychomotor work [90]. Rather, we assume that during psychomotor work the power-efficiency of the human brain is not exceeded by the processing speed of electronic computer. Hence, we assume a processing speed ratio of 10,000/1 between human and robot. However, wearing of an exoskeleton can reduce automaticity and increase the number sensory inputs needed by a cyborg worker; for example, from unexpected effects on balance [91]. Accordingly, we assume the processing time ratio for three different types of workers to be 10,000/12,000/1.

## 4. Heuristic Modelling of Effects on External Actions (*S_e_*) from Disturbances

In this section, formulae and ratios are provided for modelling effects on external action (*S_e_*) from disturbances. Heuristic ratios are applied widely in planning of production work [74,75], including for characterizing effects on production from the different attributes of different worker types [76,77] to determine the most suitable mix of worker types [78,79]. The ratios stated in this section are novel as they are for different effects on *S_e_* in production work due to different attributes of human, cyborg, and robot workers.

### 4.1. Formulae

External action (*S_e_*) is described in Equation (29) [92], where *KE(t)* denotes kinetic energy and *PE(t)* denotes potential energy of an actuator, such as human hand or robot arm. *KE* and *PE* are both functions of time

(29) $$S_{e}=\int_{t_{1}}^{t_{2}}\left[KE\left(t\right)-PE\left(t\right)\right]dt$$

Optimal biomechanical or mechatronic action minimizes the external action *S_e_*. Additional action due to disturbance can be modelled using the total variation. Resultant new motion may increase the action integrand, increase of completion time, or both. Thus, the upper integration limit (completion time) is no longer a fixed point. It is undetermined because, in general, we do not know the final value of action or completion time. For that reason, we need to determine the total variation of the variational problem with undetermined ending point. To begin the modelling, we can consider the motion of a body of mass *m* (e.g., a product component such as a car part) near the surface of the earth. Let (*u, v*) be coordinates parallel to the surface of the earth and the height *w* above the surface of the earth. Let external action to be minimized be

(30) $$S_{e}\left[u,v,w\right]=\int_{t_{1}}^{t_{2}}\left[KE\left(t,\dot{u},\dot{v,}\dot{w}\right)-PE\left(t,w\right)\right]dt$$

where

(31) $$KE\left(t,\dot{u},\dot{v,}\dot{w}\right)=\frac{m}{2}\left[{\left(\frac{du}{dt}\right)}^{2}+{\left(\frac{dv}{dt}\right)}^{2}+{\left(\frac{dw}{dt}\right)}^{2}\right]$$

and,

(32) PE(t,w)=mgw.

where g is gravitational acceleration.

Here, the triple (*u,v,w*) is an extremal such that

(33) $$\begin{array}{cc} u\left(t_{1}\right)=u_{1} & u\left(t_{2}\right)=u_{2} \end{array}$$

(34) $$\begin{array}{cc} v\left(t_{1}\right)=v_{1} & v\left(t_{2}\right)=v_{2} \end{array}$$

(35) $$\begin{array}{cc} w\left(t_{1}\right)=w_{1} & w\left(t_{2}\right)=w_{2} \end{array}$$

Suppose that, due to disturbance, the actual motion results in another action

(36) $$u^{*}=u+\xi_{u}$$

(37) $$v^{*}=v+\xi_{v}$$

(38) $$w^{*}=w+\xi_{w}$$

defined over $\left(t_{1},t_{2}+\delta t\right)$ so that

(39) $$S_{e}\left[u+\xi_{u},v+\xi_{v},w+\xi_{w}\right]=\int_{t_{1}}^{t_{2}+\delta t}\left[KE\left(t,\dot{u}+\dot{\xi_{u}},\dot{v}+\dot{\xi_{v}},\dot{w}+\dot{\xi_{w}}\right)-PE\left(t,w+\xi_{w}\right)\right]dt$$

Thus, the total variation due to the chokes arising from disturbance is

(40) $$\begin{array}{lll} \Delta S_{e} & = & S_{e}\left[u+\xi_{u},v+\xi_{v},w+\xi_{w}\right]-S_{e}\left[u,v,w\right]\\ & = & \int_{t_{1}}^{t_{2}+\delta t}\left[KE\left(t,\dot{u}+\dot{\xi_{u}},\dot{v}+\dot{\xi_{v}},\dot{w}+\dot{\xi_{w}}\right)-PE\left(t,w+\xi_{w}\right)\right]dt-\int_{t_{1}}^{t_{2}}\left[KE\left(t,\dot{u},\dot{v,}\dot{w}\right)-PE\left(t,w\right)\right]dt \end{array}$$

We have so far demonstrated how to determine the *S_e_* of the undisturbed state (29) and the *S_e_* of the disturbed state (39). Since the environment evolves between undisturbed and disturbed state, as shown in Figure 3, the mean value of *S_e_* can be determined by summing respective external actions with weights $\rho_{f}$ and $\rho_{c}$, that is

(41) $${\bar{S}}_{e}=\rho_{f}S_{e}\left[u,v,w\right]+\rho_{c}S_{e}\left[u+\xi_{u},v+\xi_{v},w+\xi_{w}\right].$$

From Equation (40), we obtain

$$S_{e}\left[u+\xi_{u},v+\xi_{v},w+\xi_{w}\right]=S_{e}\left[u,v,w\right]+\;\Delta S_{e}$$

Thus

(42) $$\begin{array}{c} {\bar{S}}_{e}=\rho_{f}S_{e}\left[u,v,w\right]+\rho_{c}S_{e}\left[u+\xi_{u},v+\xi_{v},w+\xi_{w}\right]\\ =\rho_{f}S_{e}\left[u,v,w\right]+\rho_{c}S_{e}\left[u,v,w\right]+\rho_{c}\Delta S_{e} \end{array}$$

or

(43) $${\bar{S}}_{e}=\left(\rho_{f}+\rho_{c}\right)S_{e}\left[u,v,w\right]+\rho_{c}\Delta S_{e}=S_{e}\left[u,v,w\right]+\rho_{c}\Delta S_{e}.$$

because $\left(\rho_{f}+\rho_{c}\right)=1$.

The focus of this modelling is differences between the number of microstates [22] associated with different workers’ actions to concluding u,v,w. Stated simply,$\;\Delta S_{e}$ equals number of microstates multiplied by energy consumption per microstate. Following disturbance, the number of microstates in action will deviate from the optimal, and often by different amounts for different worker types. We focus upon the microstates of different worker’s actions to concluding u,v,w, because concluding u,v,w will be the same for all types of workers. For example, u,v for the end of a bolt determine u,v for the nut to be put onto the bolt. In other words, choke-points arise from disturbances that are bounded. Therefore, a bounded disturbance in the production of a car does not lead to the production of something else, such as a bicycle. Rather, the car is still made in accordance with the car specification, but it is made with increased action because of the bounded disturbance [18,19]. Similarly, w is largely determined by the work irrespective of the type of worker. For example, no type of worker would raise a light hammer high into the air above its head in order to tap a small pin into a small glazing bead. Rather, the hammer will be raised only a little above the pin. Conversely, no type of worker would raise a heavy hammer only a little to drive a fence post into the ground. Rather, the heavy hammer will be lifted high. In both cases the concluding w for the hammer is the w of the top of what is being hammered. Hence, concluding u,v,w can be assumed to be the same for all types of workers. The difference between them is the number of microstates associated with the actions that they work through towards concluding u,v,w.

**Figure 4:**
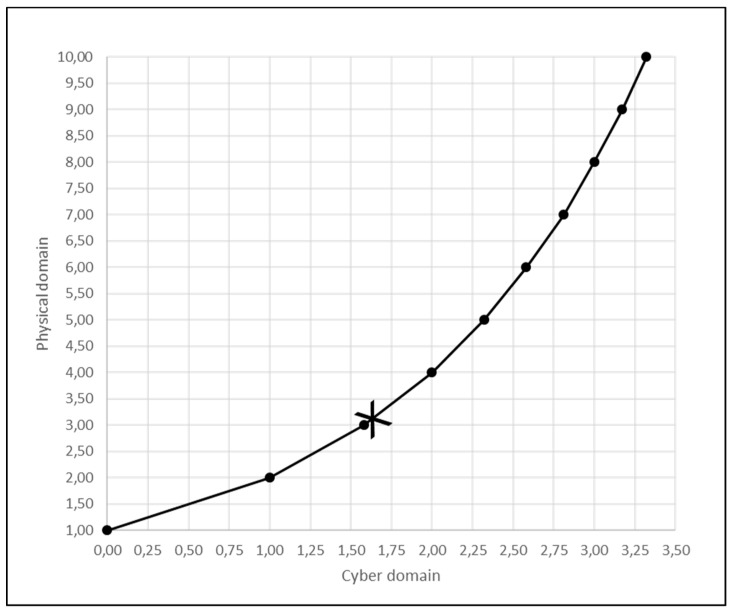
Task complexity in cyber domain and physical domain.

As is appropriate for production systems that combine the digital and the physical [93,94,95,96], we express work complexity as corresponding number of microstates in the cyber domain and in the physical domain [20]. For example, as shown in Figure 4, if task complexity in the cyber domain is 1.65, task complexity in the physical domain is 3.14, compared to the zero complexity of there being only one way to carry out the task [20]. The co-ordinates of the ten points in graph in Figure 4 mark the correspondence between task complexity in the cyber domain and task complexity in the physical domain. In particular: 0 to 1; 1 to 2; 1.58 to 3; 2 to 4; 2.32 to 5; 2.58 to 6; 2.81 to 7; 3.00 to 8; 3.17 to 9; and 3.32 to 10.

Correspondence between modelling of information (cyber domain) and of mechanics (physical domain) in terms of microstates has already been defined by others [20], and has the advantage of enabling modelling across cyber-physical systems involving different type workers. In general, entropy is given by the logarithm of the possible number of microstates. For example, 1.58 bits in the cyber domain implies 2^1.58^, which equals 3 microstates in the physical domain. Similarly, 3.32 in the cyber domain implies 2^3.32^, which equals 10 in the physical domain. The number of microstates in the physical domain is 2 to the power of entropy in the cyber domain because entropy in the cyber domain refers to bits, which is a binary random variable that is 0 or 1 with equal probability, i.e., information-theoretic entropy has two as a base [20].

In our modelling there is no difference between *S_e_* for human worker and robot worker in flow state. This is because the effects of higher robot mass (m) and lower robot agility can be reduced through application of sophisticated motion planning techniques to optimize robot action [97]. Also in our modelling, there is no difference in *S_e_* between human, robot, and cyborg workers in flow state. This is because the deployment of cyborg technologies, such as exoskeletons, in production work is also subject to application of multiple techniques for motion optimization [98]. Furthermore, it is not intended that exoskeletons cause fundamental change motions that are natural to humans, or have been optimized through the previous application of techniques, such as job design [99]. 

### 4.2. Ratios

However, the relative mass and maneuverability of different worker types can have different effects on their *S_e_* outside of the flow state. Research into robot motion provides insights into the range and consequences for *S_e_* from different robot motions involved in production work. For example, robot *S_e_* is optimized at speeds between 50 and 100 percent that avoid large accelerations and decelerations. Also, robot *S_e_* is optimized when payloads do not exceed 80 percent of maximum. By contrast, robot *S_e_* can be expected to be highest when robots have to slow down to maneuver to get into position, and when robot arms have to operate slowly. In addition, friction at robot joints can increase *S_e_* by up to 20 percent depending upon to what extent the robot has “warmed up” [100]. Sophisticated motion planning techniques to optimize robot action are not readily applicable to re-positioning and to re-performing concluding u,v,w following choke. Thus, there is little potential to reduce the effects of robots’ higher mass on *S_e_*. Also, there can be friction following the robot having been stationary in the choke state. In addition, pressure to make up for time lost in choke state can lead to increased speeds with larger accelerations and decelerations. Furthermore, payloads may be increased in efforts to try to catch up lost time. Moreover, there can be repeated maneuvering as the robot moves backwards and forwards to get into new positions, where robot arms will perform work more slowly due to the need for precision in rework. Overall, it can be expected that the higher mass (m) and lower maneuverability of robots will affect *S_e_* in re-positioning and rework.

Compared to a human worker, a robot worker can be expected to have at least 50 percent higher mass. For example, the widely publicized two-armed robot Baxter is six feet three inches tall weighing 306 pounds (139 kg) [101]. By contrast, the 2016 averages for humans in the United States of America (USA) are five feet four inches tall, weighing 170 pounds or 77 kg for females and five feet nine inches tall weighing 198 pounds or 90 kg for males [102]. In addition to lower mass of at least 50 percent, human workers have natural motion advantages for *S_e_* in complex actions. In particular, human workers have natural agility across gross and fine motor actions, because the human skeletomuscular system is evolved for dynamic movement [103,104]. Accordingly, we assume a conservative motion ratio of 1/1.5 for human worker to robot worker. For cyborg workers, heavier exoskeletons enable handling of heavier payloads. However, the consequent increase in mass brings increased *S_e_*. At the same time, heavier exoskeletons reduce agility. Moreover, research indicates that exoskeletons can reduce *S_e_* when they are worn for one type of motion, for which they have been specifically designed. However, positive effects from wearing an exoskeleton for one type of motion, such as lifting, can switch to negative effects for a related motion, such as carrying [105,106]. Thus, we assume a conservative motion ratio of 1/1.1 for human to cyborg worker in repositioning and rework, which involves a wider range of motions than positioning and performing in the flow state.

## 5. Examples

In this section, examples are provided of application of the heuristic framework explained above in Section 2, Section 3 and Section 4. As is appropriate in rule-of-thumb heuristics, we perform qualitative analysis of *S_int_* and *S_e_* that includes the ratios described above to express fundamental differences between worker types. *S_int_* and *S_e_* are considered separately because of the huge difference in their orders of magnitude. In particular, *S_int_* is miniscule compared to *S_e_*. Hence, it is not practical to sum *S_int_* and *S_e_* for individual calculations because one worker type’s much better *S_int_* will always be overridden by another worker type’s slightly better *S_e_*. However, *S_int_* is not trivial because the combined total of *S_int_* across millions of workplaces throughout the world is huge. Accordingly, it is important to compare *S_int_* for different worker types.

The examples follow four steps: calculate $\rho_{f}h\left(X,\;Y,\;Z\right)$ and $\rho_{c}h\left(X^{*},Y^{*},Z^{*}\right)$; calculate *S_int_*; calculate *S_e_*; compare *S_int_* and *S_e_* between worker types to identify worker type with lowest *S*. The first step involves consideration of task complexity as expressed with probability mass functions. This is simply done when the number and probability of different ways of working are expressed as fractions, such as 4/6, 1/6, 1/6 [5]. For example, it can be done when an expert team meets to discuss several alternative production investment options but the exact performance of each option cannot be predicted accurately in advance. Such potential production options are common and include building entirely new factories and deploying emerging cyborg technologies. Here, as is common with rule-of-thumb heuristics [41,42], the expert team comprising production engineer, production manager, financial manager, etc., bring their specialist knowledge to bear when considering alternative production options. In the first step, this expertise is applied to express the complexity of positioning, performing, and perfecting actions in a task before disturbance (X,Y,Z) and after disturbance $\left(X^{*},Y^{*},Z^{*}\right).\;$In the second step, the rule-of-thumb ratios are applied to the calculation of *S_int_*. In the third step, the rule-of-thumb ratios are applied to the calculation of *S_e_*. The fourth step is to compare *S_int_* and *S_e_* between worker types.

Table 3 provides summary of calculations in accordance with Equation (25)

$$h\left(Q\right)=\rho_{f}h\left(X,Y,Z\right)+\rho_{c}h\left(X^{*},Y^{*},Z^{*}\right)-\rho_{f}\log_{2}\rho_{f}-\rho_{c}\log_{2}\rho_{c}$$

*S_int_* for human worker can be calculated with Equation (28) as follows

$$S_{int}=c_{H}{\left[h\left(Q\right)\right]}^{2}\left(t_{2}-t_{1}\right)$$

(1 × 5.32) × (5.32 × (10000 − 0))

5.32 × 53200

283024

*S_int_* for cyborg worker can be calculated with Equation (28) as follows

(1.2 × 5.32) × (5.32 × (12000 − 0))

6.38 × 76608

488759

*S_int_* for robot worker can be calculated with Equation (28) as follows

(10000 × 5.32) × (5.32 × (1 − 0))

53200 × 5.32

283024

In this example, where *h(Q)* is the same for all types of workers, *S_int_* is equal for human worker and robot worker, because the differences in their respective ratios for power consumption (1/10,000) and processing time (10,000/1) balance each other. *S_i_* is higher for the cyborg worker due to the higher ratio for power consumption (1/1.2/10,000) and for processing time (10,000/12,000/1).

**Figure 5:**
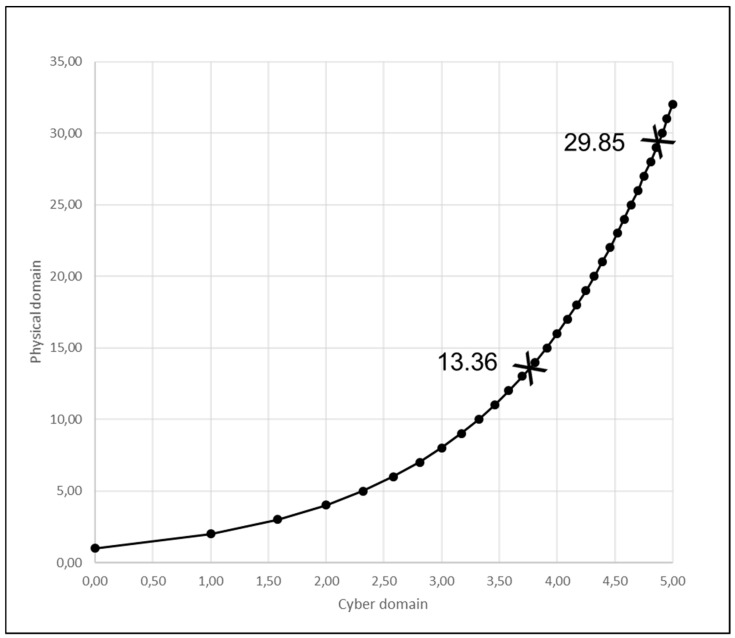
Number of microstates in actions to concluding u,v,w before and after disturbance.

When calculating ${\bar{S}}_{e}$ we add S_e_ for work carried out in flow state up to the additional S_e_ involved in extra (Δ) work arising from the disturbance. For human worker ${\bar{S}}_{e}\;$can be calculated for Equation (43) with reference to the chart in Figure 5 below as follows:

$${\bar{S}}_{e}=S_{e}\left[u,v,w\right]+\rho_{c}\Delta S_{e}.$$

2^(3.74) + 0.5[2^(4.90) − 2^(3.74)]

13.36 + 0.5(29.85 − 13.36)

13.36 + 8.245

21.61

${\bar{S}}_{e}$ for cyborg worker can be calculated for Equation (43) with reference to Figure 5 below as follows

2^(3.74) + 0.5[2^(4.90) × 1.1 − 2^(3.74)]

13.36 + 0.5[(29.85 × 1.1) − 13.36] 

13.36 + 0.5[32.84 − 13.36]

13.36 + 0.5[19.47]

13.36 + 9.738

23.10

${\bar{S}}_{e}$ for robot worker can be calculated for Equation (43) with reference to Figure 5 below as follows

2^(3.74) + 0.5[2^(4.90) × 1.5 − 2^(3.74)]

13.36 + 0.5[(29.85 × 1.5) − 13.36] 

13.36 + 0.5[44.78 − 13.36]

13.36 + 0.5[31.41]

13.36 + 15.71

29.07

In this example, where work complexity is the same for all types of workers, *S_e_* is lowest for the human worker and highest for the robot worker due to their respective motion ratios (1/1.5).

However, it cannot be assumed that work complexity will always be the same for all worker types. Rather, work settings, work composition, and work uncertainty need to be engineered to minimize the effects from disturbances for different types of workers.

For example, a human worker’s re-positioning actions can involve automaticity in the highly flexible deployment of general psychomotor abilities. Re-positioning actions can involve known solutions, comprising gross and fine general psychomotor abilities, which enable the human worker to move forward fluidly to get into position. For a cyborg worker, such as a human wearing a motorized exoskeleton, increased strength and endurance from wearable mechatronics can be offset by reduced biomechanical flexibility. For example, the exoskeleton framework can restrict medial and lateral rotation [107]. This can lead to the cyborg worker moving forward into position with more separate discrete motions than the human worker. For robot workers, re-positioning actions involve breaking down a desired movement into discrete motions that satisfy movement constraints while seeking to optimize movement. However, robot chassis do not have the flexibility of the human body nor the human body when restricted by the wearing of an exoskeleton. For example, as formalized in standard robot motion challenges, such as The Piano Mover’s Problem, robot maneuvering to get into position within workspaces that include corners involves more choke iterations than continuous flow, as robots move backwards as well as forwards in order to make adjustments in direction [108]. Accordingly, from one disturbance there will be fewest individual discrete positioning actions, and most continuous fluid positioning actions, from the human worker. The number of re-positioning iterations is influenced by work setting. Consider, for example, the disturbance of an agricultural worker falling down on sloping ground that has become slippery and undulating from combinations of heavy rainfall and worker traffic. A human worker can readily deploy general psychomotor ability known solutions developed through play and sport while growing up. Accordingly, few iterations of applying known solutions are required. Iterations of applying known solutions will be higher for the cyborg worker when there are misalignments between the human body and the exoskeleton framework, which have not been experienced before. As summarized in Table 4, for the robot worker, there will be more iterations because of the number of discrete actions needed to reestablish an upright position on slippery sloping ground, where the use of mechanical claws, etc., can be counterproductive, as they churn up the soft ground making it less stable. This example illustrates the need to focus engineering work, such as the engineering of work settings for robot implementations, on reducing effects from disturbances. If necessary engineering work is neither feasible nor viable, then worker types should not be those for which disturbances will lead to large increases of *S_int_ and S_e_*. As shown in Table 4, the differences between worker types can be so large that detailed calculations of *S_int_* and *S_e_* are not required. 

For workers, performing actions involve applying known solutions comprising different levels of psychomotor work skills schema. The higher the level of known solution that can be applied, the lower can be the number of iterations required following disturbance because the final deviation from optimal solution. However, some types of psychomotor work inevitably require application of low level work skills schema.

For example, clothing production involves shaping materials through cutting and forming parts through sewing. This is because the production of soft products such as clothing is not suited to parts consolidation techniques, which involve integration of many loose small parts into a few large assemblies [109]. Similarly, rework of soft products involves low-level work skills schema. For example, incorrect stitching needs to be pulled out and resewn. The number of iterations of rework required by different types of workers is influenced by work composition. A human worker will work with cloths that have long-established textile properties, including unpredictable distortions throughout manufacturing. By contrast, cloths used by robot workers can be stiffened temporarily by being drenched in a liquid thermoplastic solution. Robots worker, which lack human dexterity in handling unpredictable textile deformations, can then sew and shape the stiffened textile. When manufacturing is complete, the cloth in the completed apparel is washed with warm water and becomes soft once again. As well as simplifying sewing, the temporary rigidity of the textile can simplify resewing by eliminating unpredictable distortions of the textile [110]. Hence, as summarized in Table 5, it can be anticipated that the human worker will have more iterations of applying known solutions than the robot worker following disturbance.

As shown in Table 5, the differences between worker types may require detailed calculations. As power consumption ratio 1/10,000 and processing time ratio 10,000/1 for human worker to robot worker balance each other, there is no need to calculate *S_i_* for this example where 0.54 and 1.00 are clearly lower than 1.37 and 2.75. However, the 1/1.5 motion ratio for human worker to robot worker necessitates calculation of ${\bar{S}}_{e}$.

${\bar{S}}_{e}\;$for human worker can be calculated for Equation (43) as follows:

$${\bar{S}}_{e}=S_{e}\left[u,v,w\right]+\rho_{c}\Delta S_{e}.$$

2^(1.37) + 0.5[2^(2.75) − 2^(1.37)]

2.58 + 0.5(6.73 − 2.58)

2.58 + 2.07

4.65

${\bar{S}}_{e}\;$for robot worker can be calculated for Equation (43) as follows

2^(0.54) + 0.5[2^(1.00) × 1.5 − 2^(0.54)]

1.45 + 0.5[(2 × 1.5) − 1.45] 

1.45 + 0.5[3 − 1.45]

1.45 + 0.5[1.55]

1.45 + 0.72

2.17

This example illustrates that robot workers can have lower *S_e_* despite the 1/1.5 motion ratio of human worker to robot worker. Cyborg workers are not considered in this example because cyborg technologies, such as powered gloves, are not suited to the fine psychomotor skills involved in resewing.

With regard to perfecting actions, psychomotor work skills can be considered across a continuum from closed to open. An example of a closed psychomotor work skills is typing in office work, which involves development of routine expertise in one manual skill with one tool and one type of material. An example of an open skill is any of the craft skills in construction work that involve developing adaptive expertise encompassing many different skills with many different tools and many different types of materials. In between the most closed and most open work skills are those skills acquired in factory work through job enlargement, for example in order to undertake several different tasks in car assembly work [111]. For workers with expertise in closed skills, repositioning and rework involve little need for the re-perfecting of their skills. For workers with expertise in open skills, by contrast, repositioning and rework bring opportunities to re-perfect their skills through the implicit natural processes of psychomotor skills learning. However, stress caused by disturbance followed by iterations of repositioning and reworking can inhibit natural processes of psychomotor skills learning. In particular, acute stress activates selective molecules called corticotropin-releasing hormones, which disrupt the process by which the brain collects and stores memories [112]. For robot workers, re-perfecting skills based on iterations of repositioning and rework is fundamentally difficult when the specific features of the disturbance, repositioning, and rework occur only once. In particular, reprogramming, deep learning, and learning from human demonstration are all of limited usefulness when disturbances and their consequences are unpredictable, which they inevitably are in open psychomotor skills work [113]. Thus, as summarized in Table 6, it is realistic to assume that there can be many possible re-perfecting actions for human workers, while re-perfecting actions for robot workers will not take place. Consequently, it can be anticipated that there will be few long-term reductions in the number of iterations of repositioning and rework following disturbances. This is the situation in industries deploying open psychomotor skills, such as construction, which suffer from persistent productivity and quality problems [114]. By contrast, cyborg workers can use implanted or wearable technologies to make explicit that which they may have learnt from repositioning and rework. For example, this can be in the form of making video recordings that can be digitally labelled with semantic tags for subsequent retrieval. However, it can be anticipated that it is low level psychomotor work skill schema that will be re-perfected. This is because the higher the level of the schema, for example, reworking of an entire specific product, the less transferable the schema is to other perturbed tasks in open psychomotor work skills [115]. Nonetheless, modelling should be focused upon the effects of disturbances on cyborg workers.

As these examples illustrate, modelling of effects on *S* from disturbances should be informed from the outset by understanding of the limitations of different types of workers in relation to different types of work. Moreover, these examples highlight that the fundamental question in determining how work can be carried out with least action is how can work be engineered to reduce the number of different ways in which different worker types can undertake positioning and repositioning, performing and reperforming, and perfecting and reperfecting? In simple terms, there are no skill shortages where there is zero situated entropy because there is only one way that work can be carried out and there is no uncertainty about what that one way is to the type of worker undertaking the work. However, the question about how to engineer for zero situated entropy needs to take into account the effects of disturbances, rather than engineer only for the flow state and ignore the occurrence of disturbances, because individual disturbances have low frequency of occurrence and low temporal predictability.

## 6. Conclusions

### 6.1. Principal Contributions

Building upon previous studies [5,6], this paper provides five further contributions to changing the perspective and increasing the objectivity through which potential investments in improving psychomotor work can be analyzed. First, a framework for heuristic modelling of disturbances and their effects is provided. In addition to PLPA and situated entropy, this heuristic framework encompasses Markov processes, the theory of perturbations, and calculus of variations. Second, formulae and ratios are provided for heuristic modelling of effects on internal action (*S_int_*) from disturbances to psychomotor work. Third, formulae and ratios are provided for heuristic modelling of effects on external action (*S_e_*). Fourth, examples are provided of modelling disturbances heuristically in psychomotor work. Fifth, formulae and examples show how task complexity can be modelled heuristically in terms of microstates across the cyber domain and the physical domain of cyber-physical systems. Overall, the study reported in this paper addresses variational aspects of PLPA. This is important because psychomotor work is beset by disturbances, and decisions about how best to deploy what types of workers needs to be informed by analyses of disturbed tasks, as well as the optimal undisturbed tasks. Production disturbances have been modelled previously in manufacturing [1,2] and in construction [3,4]. However, previous modelling has been concerned with autonomous systems [1,2,3,4], rather than production that involves interactions between different types of work and different types of workers.

The contribution of the three papers together [5,6] is to provide a heuristic framework that enables simultaneous consideration of the cyber domain and the physical domain in cyber-physical systems that combine the digital and the physical. In particular, the three papers provide detailed step-by-step explanation of how analyses of situated entropy in psychomotor work can be carried to out to guide engineering of work setting, work composition, and work uncertainty. This focus on heuristic modelling-situated entropy to inform analyses of complexity in the cyber domain and the physical domain is increasingly relevant, because there is increasing digitalization in the production of physical goods involving psychomotor work [70,71,93,94,95].

### 6.2. Implications for Practice

John von Neumann opined that mathematical models should describe phenomena from a reasonably wide area and should be simple [116]. Such mathematical models are needed to debias investment decision-making for production technologies. This is because investment decision-making is overly influenced by hype about advances in technologies, and this leads to expensive investments in new technology implementations that subsequently have to be removed from production operations because of poor performance [117,118]. Debiasing investment decision-making involves changing perspective to increase the objectivity through which potential investments are analyzed [119,120]. As shown by the examples in Section 5, the framework introduced in this paper conforms to von Neumann’s opinion and is in-keeping with the general characteristics of rule-of-thumb heuristics. Most importantly, the framework is appropriate for engineering design, where there are several alternative production options to be considered, and the exact performance of each option cannot be measured accurately in advance. This lack of performance details precludes meaningful application of simulation tools, but does not preclude application of rule-of-thumb heuristics [24,25,26,27,28,29,30,31,32,33,34,35,36,37,38,39,40,41,42,43,44].

### 6.3. Limitations and Directions for Further Research

We have used a two-state Markov model to capture the dynamic behavior of different types of workers. An essential part of the model is the so-called stationary distribution of the flow and choke states. As we pointed out in the Section 2, we assume that transition probabilities between the flow and choke state are necessary time-invariant. This assumption may not be valid in all production environments. For example, large-scale production sites with tasks performed repeatedly in an accurately controlled environment can usually be modelled with two-state Markov processes. On the other hand, small-scale production with many unique tasks and a rapidly changing environment, for example, construction of a building on a lot with many unique terrain features, rarely can be described with simple two state-Markov processes. In these cases, where the production environment is nonstationary, the information-theoretic entropy in the Shannon’s sense given by Equation (5) can lose some of its operational meaning. The extension of the proposed model to nonstationary and arbitrarily varying environments is the subject of future research.

## Figures and Tables

**Table 1:** Sources of disturbances in psychomotor work.

Theory	Variables		Sources of Disturbances Examples
Embodied cognition [14]	Worker types	Human	Fatigue errors
		Cyborg	Body/exoskeleton alignment errors
		Robot	Sensor errors
Work pragmatics [15]	Work characteristics	Setting	Weather conditions
		Composition	Natural materials
		Uncertainty	Inconsistent interfaces
Situated cognition [16]	Worker-work interactions	Positioning actions	Slipping on ground dampened by rainfall
		Performing actions	Misalignments when working natural material
		Perfecting actions	Sensing errors of unique component interfaces
Cognitive load [17]	Embodied cognitive load	Extraneous	“Surprise” of unexpected sensory input
		Intrinsic	Processing of misalignment information
		Germane	Active inference to match inputs with schema

**Table 2:** Known solutions psychomotor work.

Known Solutions	Level	Example
Psychomotor work skills schema	Reforming products interfaces	Interface between reception desk and wall
	Reassembling products	Reception desk
	Refitting sub-assemblies	Desk top
	Remaking parts	Desk drawer
	Reshaping materials	Wood
Psychomotor general ability templates	Fine: dorsiflexion	Increasing palm–inner-arm angle to place part
	Fine: palmar flexion	Decreasing palm–inner-arm angle to hold part
	Gross: medial/lateral rotation	Rotating arm closer or away from body for task
	Gross: abduction/adduction	Raising and lowering arms to reach work
	Gross: flexion/extension	Walking, standing, sitting to do work

**Table 3:** Calculation of $\rho_{f}h\left(X,\;Y,\;Z\right)$ and $\rho_{c}h\left(X^{*},Y^{*},Z^{*}\right)$.

Complexity			Disturbance	Complexity Following Disturbance		
*X*	4/6, 1/6, 1/6	1.25	work piece damaged due to sensory error	X*	1/3, 1/3, 1/3	1.58
*Y*	1/10, 1/10, 6/10, 1/10, 1/10	1.77		Y*	1/5, 1/5, 1/5, 1/5, 1/5	2.32
*Z*	4/5, 1/5	0.72		Z*	1/2, 1/2	1.00
h(X, Y, Z)	1.25 + 1.77 + 0.72	3.74		$h\left(X^{*},Y^{*},Z^{*}\right)$	1.58 + 2.32 + 1.00	4.90
*f*	0.9			*c*	0.9	
$\rho_{f}$	0.5			$\rho_{c}$	0.5	
$\rho_{f}h\left(X,\;Y,\;Z\right)$		1.87		$\rho_{c}h\left(X^{*},Y^{*},Z^{*}\right)$		2.45
h(Q) = 1.87 + 2.45 − 0.5$\log_{2}\left(0.5\right)-$ 0.5$\log_{2}\left(0.5\right)$ = 5.32						

**Table 4:** Positioning and re-positioning.

Worker Type	Positioning			Repositioning Following Disturbance		
	Complexity	Cyber	Physical	Complexity	Cyber	Physical
Human	1/6, 4/6, 1/6	1.25	2.38	1/8, 3/8, 1/8, 2/8, 1/8	2.16	4.47
Cyborg	1/6, 4/6, 1/6	1.25	2.38	1/10, 1/10, 3/10, 2/10, 2/10, 1/10	2.45	5.46
Robot	1/6, 4/6, 1/6	1.25	2.38	1/12, 1/12, 1/12, 3/12, 2/12, 1/12, 2/12, 1/12	2.85	7.21

**Table 5:** Performing and re-performing.

Worker Type	Performing			Re-performing Following Disturbance		
	Complexity	Cyber	Physical	Complexity	Cyber	Physical
Human	1/5, 3/5, 1/5	1.37	2.58	1/8, 1/8, 2/8, 1/8, 2/8, 1/8	2.75	6.73
Cyborg	n/a			n/a		
Robot	1.75/2, 0.25/2	0.54	1.45	1/2, ½	1.00	2.00

**Table 6:** Perfecting and re-perfecting.

Worker Type	Perfecting (none in Flow State)			Re-perfecting Following Disturbance		
	Complexity	Cyber	Physical	Complexity	Cyber	Physical
Human	n/a	n/a	n/a	1/25, 1/25, 1/25, 1/25, 1/25, 2/25, 4/25, 4/25, 3/25, 3/25, 1/25, 1/25, 1/25, 1/25	3.54	11.67
Cyborg	n/a	n/a	n/a	1/2, 1/4, 1/4	1.5	2.86
Robot	n/a	n/a	n/a	n/a		
